# Pre-clinical impact of higher irrigation flow rates during pulsed field ablation with a variable loop circular catheter

**DOI:** 10.1093/europace/euag120

**Published:** 2026-05-16

**Authors:** Fengwei Zou, Xiao-dong Zhang, Tara Gomez, Eric Byun, Qi Chen, Jacopo Marazzato, Marco Schiavone, Sanghamitra Mohanty, Vincenzo Mirco La Fazia, Jishu Motta, Cristian Zamora Rosales, Shubhi Pandey, Lowell Safren, Yuval Shafir, Vito Grupposo, Domingo Ynoa, Aung Lin, Dhanunjaya Lakkireddy, Andrea Natale, Nils Guttenplan, Luigi Di Biase

**Affiliations:** Montefiore-Einstein Center for Heart and Vascular Care, Montefiore Medical Center, Albert Einstein College of Medicine, 111 E 210th street, Bronx, NY, USA; Montefiore-Einstein Center for Heart and Vascular Care, Montefiore Medical Center, Albert Einstein College of Medicine, 111 E 210th street, Bronx, NY, USA; Biosense Webster Inc., part of Johnson and Johnson MedTech., Irvine, CA, USA; Biosense Webster Inc., part of Johnson and Johnson MedTech., Irvine, CA, USA; Biosense Webster Inc., part of Johnson and Johnson MedTech., Irvine, CA, USA; Montefiore-Einstein Center for Heart and Vascular Care, Montefiore Medical Center, Albert Einstein College of Medicine, 111 E 210th street, Bronx, NY, USA; Department of Clinical Electrophysiology & Cardiac Pacing, Monzino Cardiological Center, IRCCS, Milan, Italy; Texas Cardiac Arrhythmia Institute, St David's Medical Center, Austin, TX, USA; Texas Cardiac Arrhythmia Institute, St David's Medical Center, Austin, TX, USA; Montefiore-Einstein Center for Heart and Vascular Care, Montefiore Medical Center, Albert Einstein College of Medicine, 111 E 210th street, Bronx, NY, USA; Montefiore-Einstein Center for Heart and Vascular Care, Montefiore Medical Center, Albert Einstein College of Medicine, 111 E 210th street, Bronx, NY, USA; Montefiore-Einstein Center for Heart and Vascular Care, Montefiore Medical Center, Albert Einstein College of Medicine, 111 E 210th street, Bronx, NY, USA; Montefiore-Einstein Center for Heart and Vascular Care, Montefiore Medical Center, Albert Einstein College of Medicine, 111 E 210th street, Bronx, NY, USA; Montefiore-Einstein Center for Heart and Vascular Care, Montefiore Medical Center, Albert Einstein College of Medicine, 111 E 210th street, Bronx, NY, USA; Biosense Webster Inc., part of Johnson and Johnson MedTech., Irvine, CA, USA; Montefiore-Einstein Center for Heart and Vascular Care, Montefiore Medical Center, Albert Einstein College of Medicine, 111 E 210th street, Bronx, NY, USA; Montefiore-Einstein Center for Heart and Vascular Care, Montefiore Medical Center, Albert Einstein College of Medicine, 111 E 210th street, Bronx, NY, USA; Kansas City Heart Rhythm Institute, Overland Park, KS, USA; Texas Cardiac Arrhythmia Institute, St David's Medical Center, Austin, TX, USA; Montefiore-Einstein Center for Heart and Vascular Care, Montefiore Medical Center, Albert Einstein College of Medicine, 111 E 210th street, Bronx, NY, USA; Montefiore-Einstein Center for Heart and Vascular Care, Montefiore Medical Center, Albert Einstein College of Medicine, 111 E 210th street, Bronx, NY, USA

**Keywords:** VARIPULSE™, Variable loop circular catheter, Atrial fibrillation, Irrigation

## Introduction

The pulsed field ablation (PFA) variable loop circular catheter (VLCC; VARIPULSE™) has demonstrated long-term clinical safety and efficacy.^1^ VLCC was initially approved with a 4 mL/min irrigation rate. When the VLCC was first released in the US, reports of increased neurovascular events led to a suspension of use of the VLCC system. Workflow steps that mitigated root causes for thermal heating were identified, and subsequently, the 30 mL/min flow rate was introduced to prevent electrode surface temperature rise.^2^ In this preclinical study, a combination of porcine simulated use procedures and bench testing were performed to compare 30 mL/min vs. 4 mL/min irrigation for acute and terminal efficacy, tissue heating response, haemolysis, lesion depth, and microbubble formation.

## Methods

### Subjects and setup

Six Yorkshire pigs were used. Subjects were divided into control (4 mL/min irrigation, *n* = 3) and experimental (30 mL/min, *n* = 3) groups. A benchtop vegetal potato model and a ventricular myocardial model were also utilized to evaluate the biophysics of PFA lesion formation between each group.

### Ablation procedure

The ventricular lesion model was performed as previously described.^3^ To assess acute efficacy and haemolysis, simulated procedures were performed with ≥80 ablations in atrial regions (right pulmonary veins, left atrial appendage, posterior wall) using a VLCC and TRUPULSE™ Generator (Johnson and Johnson MedTech, Irvine, CA, USA). Pulmonary vein isolation (PVI) was confirmed post-ablation. Blood samples for plasma-free haemoglobin (PFH) were taken at pre- and post-ablation intervals. The VLCC was also visually inspected after delivery of 32, 48, 64, and 80 ablations in the left atrium for signs of blood coagulum or char. Animals were re-mapped on day 5 ± 1 and humanely euthanized. Necropsies evaluated collateral damage and thrombo-emboli, with gross and histopathology.

Electrode/tissue temperature measurements were assessed in a circulating saline bath similar to previously described,^4^ with the key distinction of being performed on non-perfused bovine cardiac tissue as opposed to perfused porcine thigh muscle. All ablations were conducted with the catheter placed in contact with the tissue at a preset perpendicular force of 30 g. As a comparator, the THERMOCOOL STSF catheter with 8 mL/min irrigation flow, 30 Watts power, and 30-second duration (Johnson and Johnson MedTech, Irvine, CA) was used. Fiberoptic probes measured temperature changes from baseline at the tissue surface and depths of 3 and 7 mm. Temperatures were monitored immediately after ablation and until the tissue returned to baseline; the maximum temperature was recorded.

Microbubble assessment utilized an ultrasonic device (Gampt mbH, Zappendorf, Germany) in an *in vitro* extracorporeal bypass model as previously described.^5^ Ablations were performed within a circulating saline system at ∼37°C and passed to the bubble counter device.

The data underlying this article will be shared on reasonable request to the corresponding author.

### Statistics

Two-sided *t*-test’s and multivariate ANOVAs analysed lesion depth, bubble size/count, temperature change and PFH differences between irrigation groups. *P* value <0.05 was considered significant.

## Results

**Figure 1:**
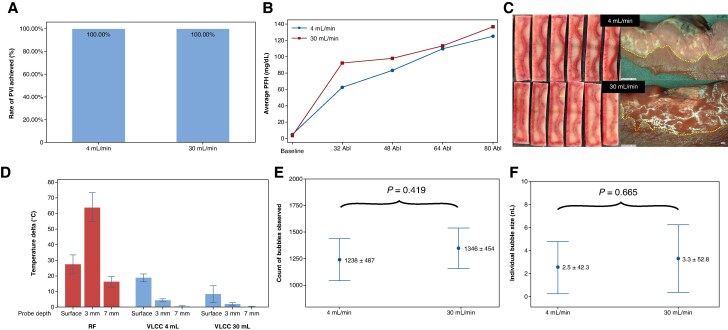
Comparisons between 4 mL/min and 30 mL/min (*A*) PVI success rates—bar graph of the rate of PVI success. (*B*) Haemolysis—plot of average plasma-free haemoglobin (PFH) at 32, 48, 64, and 80 ablations (*P* = 0481, *P* = 0.341, *P* = 0.904, *P* = 0.816, respectively). (*C*) Ventricular lesion depths—representative images of lesion depth in both the potato model (left) and ventricular model (right) (*P* = 0.280 and *P* = 0.246, respectively). Yellow arrows and lines indicate lesion depth and borders, respectively. (*D*) Temperature deltas—bar graph of ΔT at the surface, 3 mm, and for an RF catheter (left), VLCC catheter with 4 mL/min (middle), and VLCC with 30 mL/min (right) (*P* < 0.001) in a non-perfused model. (*E*) Microbubble counts—plot of counts of bubbles observed (*P* = 0.419). (*F*) Microbubble volumes—plot of bubble volumes (*P* = 0.665).

Acute and terminal (5 ± 1 days) PVI was observed in 6/6 (100%) animals across both the irrigation groups (*Figure 1A*).

VLCC visual inspection showed no significant blood coagulation in the 30 mL/min group until 64 ablations (4 times above recommended workflow), whereas blood coagulation appeared after 48 ablations in the 4 mL/min group. No char was observed.

PFH was measured from pre- and post-ablation blood draws. No significant difference in haemolysis between groups was observed (*Figure 1B*). Elevated CK values were observed in all subjects after 32 ablations (2 times clinical recommendation).

To assess lesion consistency between irrigation groups, ventricular and potato substrates were used. Lesion depth did not differ significantly between 4 and 30 mL/min groups in ventricular (3.7 ± 0.8 mm vs. 4.2 ± 0.8 mm; *P* = 0.246) or potato models (5.0 ± 0.5 mm vs. 5.2 ± 0.5 mm; *P* = 0.280; *Figure 1C*).

Using an *in vitro* circulating saline bath, the VLCC with 30 mL/min demonstrated significantly lower change in surface temperature compared with 4 mL/min (8.36 ± 5.2°C and 18.76 ± 2.35°C, respectively; *P* < 0.001; *Figure 1D*). At the 3 mm depth, temperature changed by 4.42 ± 0.69°C in the 4 mL/min group and 1.97 ± 0.85°C in the 30 mL/min group; at 7 mm depth, by 0.59 ± 0.21°C and 0.40 ± 0.13°C, respectively. Comparatively, RF with 30W power and 30 s duration demonstrated overall higher increases in temperature across all depths (surface, 3, and 7 mm) with mean temperature deltas of 27.36 ± 8.56°C, 64 ± 12.98°C, and 16.2 ± 4.94°C, respectively (*Figure 1D*). Notably, as the tissues utilized in this *in vitro* study were not perfused, these temperature deltas overestimate the thermal impact of ablation for all test groups. Notably, the 3 mm temperature data also includes *in vitro* steam pops.

Using an extracorporeal bypass model, no significant difference in mean bubble count or volume between groups was observed (*Figures* *1E* and *F*).

## Discussion

These findings suggest that a 30 mL/min irrigation rate with VLCC does not compromise PFA lesion formation, as demonstrated by consistent acute PVI and lesion formation outcomes observed in both potato and ventricular models. Other recent reports showed that 30 mL/min had a signal towards slightly deeper lesions compared with 4 mL/min lesions.^6,7^ Together with our results, evidence is consistent that 30 mL/min irrigation does not produce inferior lesions.

In this study, increasing the irrigation rate to 30 mL/min mitigates surface heating during PFA, corroborating the findings of Sauer *et al*. and Zito *et al*. that reported a reduction in electrode temperature at higher irrigation rates in potato and bovine myocardial tissue models respectively.^2,8^ Notably, despite the presence of temperature rise, the biophysics of temperature during RF ablation is markedly different from PFA, with RF energy producing sustained thermal elevation at deeper tissue depths.^4^ In contrast, PFA demonstrates minimal temperature buildup at 3 mm below the surface, albeit some thermal effects still occur due to Joule heating from the electrodes’ high-voltage energy.^2,4^ As PFA is increasingly used to target non-PV triggers such as the posterior wall, temperature rise mitigation remains important in the safety profile of PFA in prevention atrioesophageal fistulas.

Interestingly, no significant increase in bubble formation (in number or size) was observed with the higher irrigation rate. This may reflect the complex, heterogeneous nature of bubble formation during PFA, which include those from entrapped air, hydrolysis, and thermal effects.^9^ It is possible that increased irrigation may reduce bubble formation related to thermal effects (e.g. cavitation or vapour bubble generation), balancing out the overall bubble count, despite the higher flow rate.^2,9^ This suggests that higher irrigation rates may modulate the types of bubbles produced during PFA.^6^ It is also important to note that, during visual inspection of the VLCC after *in vivo* PFA delivery in the porcine LA, the 30 mL/min irrigation group showed no significant blood coagulation until 64 ablations compared to 48 ablations in the 4 mL/min group. When PFA is used to target non-PV triggers, more lesions are usually performed. Together with lower surface temperature rise, the 30 mL/min offers higher safety margin from blood coagulum formation which could be a potential source for thromboembolism. The mechanisms underlying stroke and thromboembolic events with PFA are being studied with ongoing research aimed at optimizing safety while maintaining efficacy. Nonetheless, recent reports indicate significant reduction in the rate of silent cerebral events and lesions when 30 mL/min is used as opposed to 4 mL/min^6,10^

While no significant differences in haemolysis were detected between irrigation rates here, it is unclear if higher irrigation in clinical settings could offer protective benefits, such as shunting blood from electrodes or enhancing hydration, potentially mitigating haemolytic effects as has recently been reported.^6^

Overall, this study demonstrates that utilizing the VLCC with 30 mL/min irrigation maintains comparable acute efficacy, lesion morphology, haemolysis, microbubble profiles and mitigates tissue heating, enhancing the performance profile of the procedure.

## Data Availability

Data available on request.
